# Role of Central Venous Pressure and Mean Arterial Pressure to Central Venous Pressure Ratio in Assessing Poisoning Severity and Poor Outcomes in Intensive Care Unit-Admitted Acute Aluminum Phosphide-Poisoned Patients

**DOI:** 10.1007/s12012-026-10103-0

**Published:** 2026-03-14

**Authors:** Heba Ibrahim Lashin, Mai Mohammed Mahran, Basma Adel

**Affiliations:** 1 Forensic Medicine and Clinical Toxicology Department, Faculty of Medicine, Tanta University, 6th floor, Medical Colleges complex, El-Geish Street, Tanta, 31527 Gharbia Egypt; 2 Emergency Medicine and Traumatology Department, Faculty of Medicine, Tanta University, Tanta, Egypt

**Keywords:** Central venous pressure, Mean arterial pressure, Acute poisoning, Aluminum phosphide, Outcomes, Intensive care unit

## Abstract

Aluminum phosphide (ALP) is a highly toxic pesticide endangering the healthcare system. Higher central venous pressure (CVP) values may be linked to poorer outcomes in various medical conditions. This study evaluated the role of CVP and mean arterial pressure (MAP)/CVP ratio as prognostic factors for intensive care unit (ICU) admitted acute ALP-poisoned patients. This retrospective study included 145 acutely ALP-poisoned patients who were referred to the ICU during two years. Data regarding history, clinical manifestations, laboratory investigations, and outcome were collected and stratified by quartiles (Q1-Q4) of the measured CVP during the first 6 h after admission. In-hospital mortality and need for mechanical ventilation (MV) were significantly predominant in Q3 and Q4 groups of CVP levels (89.4% and 92% & 91.5% and 96%, respectively) (*p*-values < 0.001). After vasopressors administration, survivors demonstrated a significant mean reduction in CVP measurements (–4.5 mmHg), whereas non-survivors had a mean increase (+ 0.6 mmHg) (*p* = 0.011). MAP/CVP ratio has good discriminatory power (the areas under the curve (AUCs) = 0.836 and 0.846) for predicting the need for MV and in-hospital mortality, respectively. The CVP measurement (AUCs = 0.812 and 0.782, respectively) comes in second, followed by the poisoning severity score (AUCs = 0.778 and 0.768, respectively). There was a significant decrease in survival probability in patients with CVP ≥ 25 mmHg, and MAP/CVP ratio *≤* 2.48 (*p-*values < 0.001). Lower MAP/CVP ratios and higher CVP measurements are alarming signs, warranting a higher risk of the need for MV and in-hospital mortality in acute ALP poisoning.

**Graphical Abstract:**

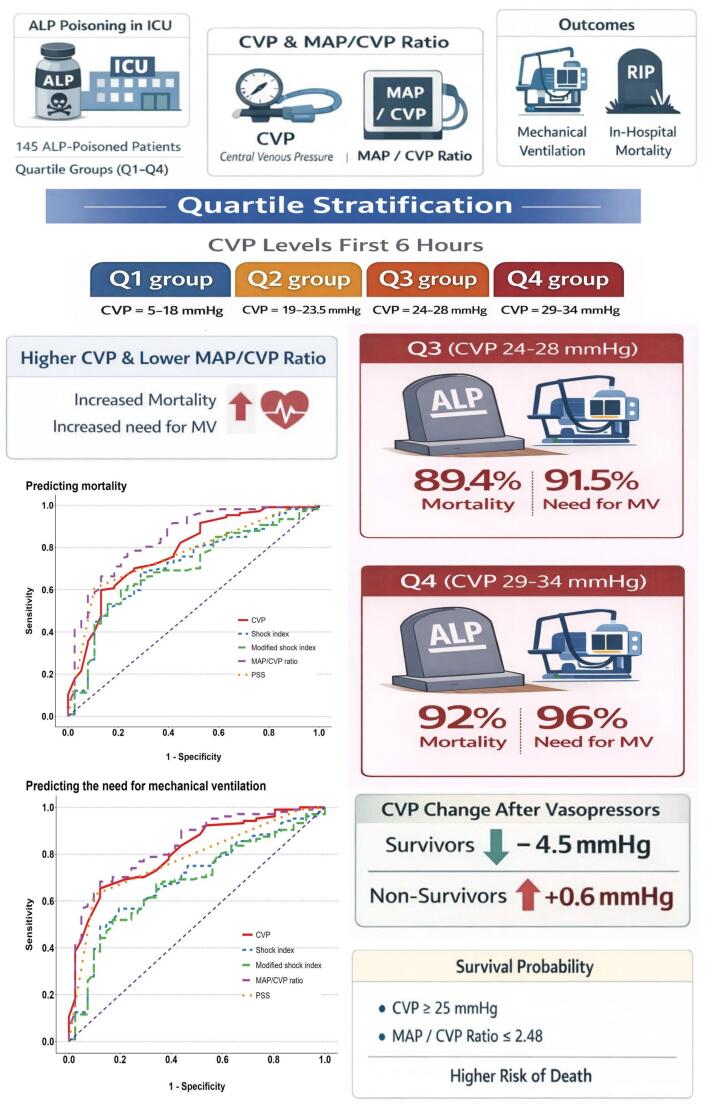

**Supplementary Information:**

The online version contains supplementary material available at 10.1007/s12012-026-10103-0.

## Introduction

Aluminum phosphide (ALP) is a highly toxic pesticide used as a grain fumigant [1]. Acute ALP poisoning is considered one of the most lethal health issues endangering the healthcare system, which has a high death rate that varies from 30% to 100% of patients [2]. Many poison centers in Egypt reported significant acute ALP poisoning rates, with an elevated incidence of mortality [3–5]. When ALP is exposed to water or gastric acidity, phosphine (PH_3_) gas is released and immediately absorbed [6]. The kidneys could metabolize phosphine and excrete it as phosphate and hypophosphite. Exhalation eliminates phosphine itself without change. Moreover, ALP can be excreted in urine unchanged [7, 8].

There are several hypothesized toxicity mechanisms, but the main one is mitochondrial malfunction linked to PH_3_’s suppression of oxidative phosphorylation, which lowers ATP synthesis and generates reactive oxygen species [9–11], Hence, the heart’s high mitochondrial content, consumption of oxygen, and metabolic activity make it the organ most susceptible to ALP poisoning [12]. As a result, cardiotoxicity and its consequences, such as refractory hypotension, severe dysrhythmia, and cardiogenic shock, explain the majority of fatalities caused by ALP poisoning [13–16]. This clearly highlights the significance of evaluating cardiac condition to assess and predict the prognosis of acute ALP-poisoned patients.

Previous studies have described ALP-induced cardiotoxicity by assessing cardiac biomarkers [17, 18], ECG [19–21], or echocardiography [22, 23]. Myocardial damage induced by ALP is associated with an increase in creatine phosphokinase (CPK), creatine kinase myocardial band (CK-MB), and Troponin-T in some cases [18, 24]. However, other researchers found these markers are unreliable as there are conflicting reports of their levels [25]. Additionally, the normal level of these enzymes could not rule out cardiac toxicity, while their raised level can confirm myocardial damage [26]. Furthermore, N-terminal pro-B-type natriuretic peptide (NT-proBNP) was found to be higher among non-survivors in contrast to the initial cardiac Troponin-I and CK-MB levels in acute ALP-poisoned patients [17]. Concerning the echocardiographic assessment, the left ventricular and intraventricular septal hypokinesia were the main abnormal findings in acute ALP poisoning. Returned to this, up to half of acute ALP-poisoned patients showed reduced ejection fraction over the first four days after poisoning [22, 23]. Therefore, future studies to determine the roles of other novel markers are needed to predict ALP-induced heart damage and to prevent the progression of poisoning through appropriate management strategies [6].

Central venous pressure (CVP) is the pressure measured in the superior vena cava or the right atrium and, to some degree, the left ventricular preload, indicating measurement of venous return and right ventricular performance [27]. The CVP measurement may therefore aid in the fluid management [28] and indicate the patient’s cardiocirculatory condition [29]. Hence, the insertion of a central venous catheter into the superior vena cava is highly recommended as an optimal standard care for critically ill patients [30]. According to several recent studies, higher or lower CVP levels can have predictive values [31–33]. Higher CVP values may be linked to poorer outcomes for patients undergoing cardiopulmonary bypass surgery [34], acute kidney injury [35], cardiac surgery [36], and critically ill patients [27]. A controlled-lowered CVP can decrease blood loss during hepatectomy [37].

Mean arterial pressure (MAP) represents a substitute for systemic vascular resistance as well as systolic and diastolic blood pressures. Left ventricular end-diastolic pressure is also impacted by MAP, especially in patients who have systolic dysfunction layered on top of excessive diastolic dysfunction [38, 39]. Hence, CVP serves as a surrogate for both right ventricular function and volume status [27]. As a result, the MAP/CVP ratio combines the functions of the left and right ventricles into a single parameter that reflects both. In addition, after the placement of a left ventricular assist device, the MAP/CVP ratio has been studied as a significant predictor of right ventricular failure [40].

In general, patients with toxic cardiac manifestations may have a higher chance of survival if they receive early supportive care [41]. Therefore, it is necessary to identify those patients quickly. Moreover, CVP measurement is considered an alarming sign for these patients in the critical care setting [42]. To the best of our knowledge, no previous studies have investigated the use of CVP measurements for early prediction of poor outcomes after acute ALP poisoning. Considering the high lethality of ALP poisoning with the absence of a specific antidote and the rising importance of measurement and follow up of CVP in critically ill patients, hence, this study was the first one aimed to evaluate the role of the CVP and MAP/CVP ratio as novel prognostic parameters for assessing poisoning severity and for early prediction of the need for mechanical ventilation (MV) and in-hospital mortality in intensive care unit-admitted acute ALP-poisoned patients.

## Patients and Methods

### Study Design and Setting

This retrospective cross-sectional study was conducted on the acutely ALP-poisoned patients admitted to Tanta University Poison Control Center (TUPCC) who were referred to the intensive care unit. The current study involved the medical records of the patients admitted during the period from the start of February 2023 to the end of January 2025. This Egyptian tertiary healthcare Center serves the Gharbia governate and the neighbouring governates in and around the Delta region that lack this medical service. Moreover, these areas are among the most densely populated places in Egypt [43].

### Sampling

Sample size was calculated using the R Statistical language (version 4.4.2) [44]. First, the sample size was calculated for receiver operating characteristic (ROC) curve analysis for individual indices for the primary outcome (mortality) using the package pROC (version 1.18.5) [45]. One ROC curve power calculation was performed, assuming an area under the curve (AUC) of 0.7 or higher, an alpha-level of 0.05, a power of 90%, and a ratio of control to cases of 0.43 for the outcome of mortality based on the incidence of mortality in ALP of 70% or more as reported by Anand et al. [46]. The minimal number was 80 cases and 34 controls (a total of 114 patients).

Second, we calculated the sample size for the correlation between the measured indices and LOS or vasopressor dose using the pwr package version 1.3-0 [47]. Assuming a correlation coefficient of at least 0.3, an alpha level of 0.05, a power of 80%, and a two-sided alternative hypothesis, the minimal required sample size was 85 patients. The final conclusion was to include the larger of the two estimates, that is, 114 patients at least. However, during the study period, a further 31 patients met the inclusion criteria and were enrolled in this study analysis to reach a sample of 145 patients and increase the study’s power.

### Inclusion Criteria

The current study included all acutely ALP-poisoned patients aged 14 and above of both genders who were admitted to TUPCC during the study period and referred to the ICU with complete medical records, including available CVP measurement records. Diagnosis of acute ALP poisoning depended upon the history given by the patient, or his/her relatives if the patient was incompetent, requesting the container if available, the presence of clinical symptoms and signs suggested exposure to ALP, but not other agents or pathological conditions, and performing a silver nitrate test on the gastric aspirate to detect phosphine gas. Additionally, acute ALP poisoning was diagnosed based on the International Classification of Diseases code T60, which refers to the toxic effect of pesticides [48].

### Exclusion Criteria

**Fig. 1:**
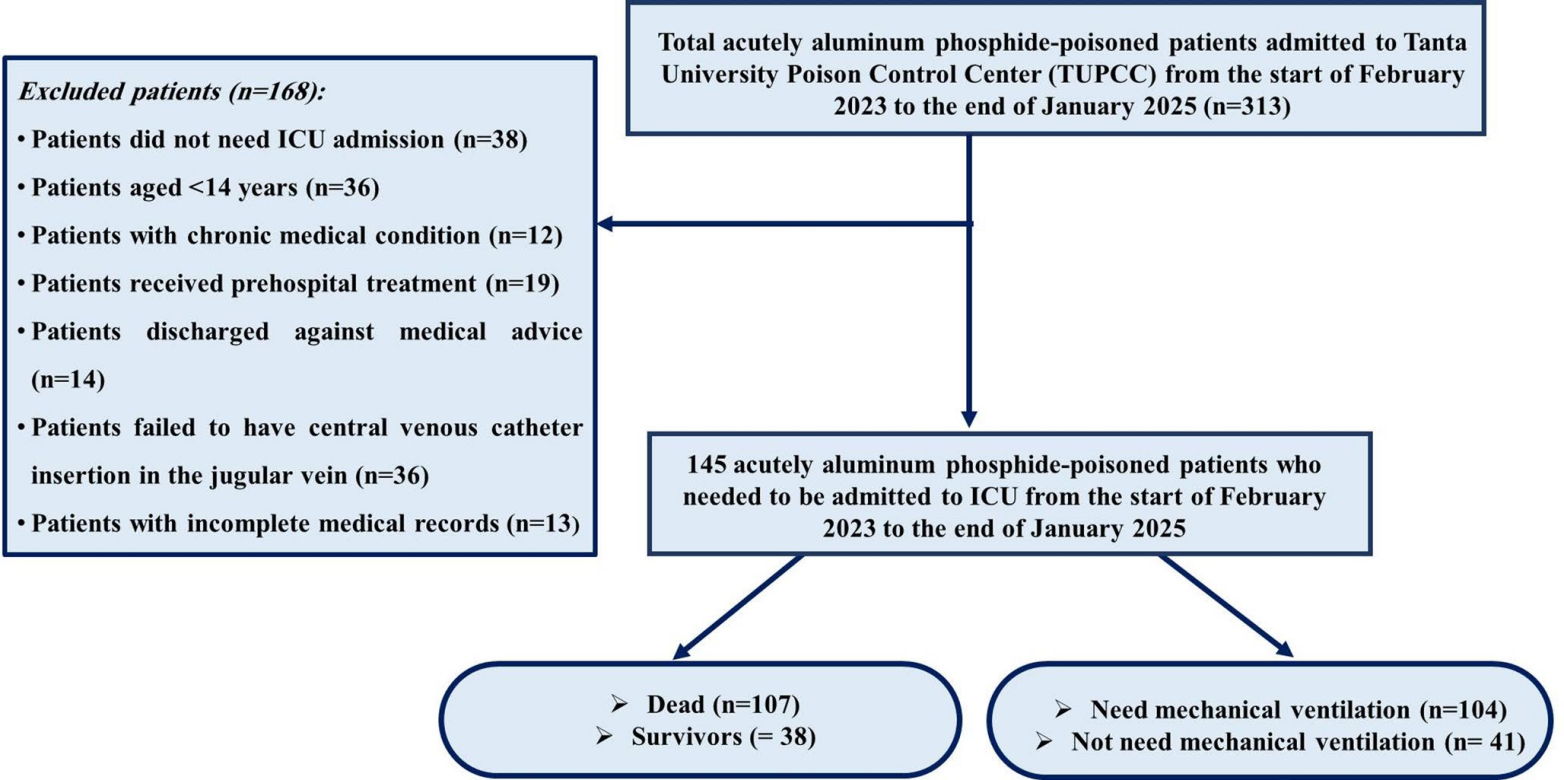
Flowchart of eligibility criteria of patients included in this study

Patients under 14 years or those who failed to have a central venous catheter in the jugular vein were excluded. Moreover, patients who had chronic medical conditions, such as neurological, cardiac, hepatic, respiratory, or renal diseases, were excluded. In addition, patients who received pre-hospital treatment or were discharged against medical advice were not included in the study. Also, cases with incomplete medical records were excluded as well. The recruitment process for patients enrolled in the present study is illustrated in Fig. 1.

### Data Collection Tool

A patient sheet form, which included the following information, was extracted from each medical record to fulfill the eligibility criteria:

#### Demographics and Toxicological History

The patient’s demographics, including age and sex, were conveyed. Toxicological history, including the amount, route, mode of poisoning and the delayed time from exposure until receiving emergency treatment, was reported.

#### Clinical Assessment

The patient’s clinical manifestations and the pupil condition upon admission were reported from the medical records of the included patients. Furthermore, the values of the vital signs at admission were extracted from the patient’s records, including systolic blood pressure (SBP), diastolic blood pressure (DBP), pulse rate, respiratory rate, oxygen (O_2_) saturation, and axillary temperature. In addition, authors calculated the MAP ([SBP-DBP]/3 + DBP), shock index (SI) (heart rate/SBP), and the modified shock index (MSI) (heart rate/MAP) [49] for each studied patient. All available CVP measurements recorded during the hospital stay were extracted. The MAP to CVP ratio was calculated for each subject using the following formula: MAP to CVP ratio = MAP/CVP [40].

The conscious level assessed by using the Glasgow Coma Scale (GCS) [50], and the Poison Severity Score (PSS) as reported by the attending toxicologist on admission were documented. Patients were categorized according to PSS into three grades: mild, moderate, and severe signs of poisoning [51]. Additionally, any abnormal electrocardiography (ECG) findings were reported.

#### Laboratory Investigations and Therapeutic Regimens

The routine laboratory investigations on admission for the enrolled patients were extracted, including measured arterial blood gases, random blood glucose levels, serum sodium, potassium & magnesium, Glucose/Potassium (Glu/K) ratio, liver transaminases, and serum urea & creatinine levels. Moreover, Complete Blood Count (CBC) values, including the hemoglobin, leucocytes, and platelet counts, were extracted.

Moreover, the total doses of the vasopressors administered to maintain the patients’ hemodynamic stability were recorded. According to the TUPCC regimen adopted from international guidelines [28, 52], acute ALP-poisoned patients may require intravenous fluid administration and vasopressors to maintain hemodynamic stability, with monitoring of the patients’ CVP level to keep it within the normal physiological limits. Additionally, the need for MV, the length of hospital stay, and in-hospital mortality were reported.

### Outcomes

Mortality was considered the primary outcome, while the need for MV, the amount of vasopressors administered, and the duration of hospital stay were secondary outcomes of this study.

### Data Analysis

Analyses were conducted using the R Statistical language (version 4.5.0) [44]. Normality of distribution was assessed using the Shapiro-Wilk test and the Q-Q plots. Continuous numerical variables following a normal distribution were summarized using the mean, standard deviation (SD), and the range (minimum to maximum values). For numerical variables that did not follow a normal distribution, the median and interquartile range (IQR, 25th–75th percentiles) were used.

Baseline characteristics were stratified by quartiles of the measured CVP during the first 6 h after the ICU admission: Q1 group (CVP = 5–18 mmHg), Q2 group (19–23.5.5 mmHg), Q3 group (24–28 mmHg), and Q4 group (29–34 mmHg). Comparisons were done using the one-way analysis of variance (ANOVA) for normally distributed variables, followed by a post hoc test if significant (Tukey’s test or Games-Howell test based on Homogeneity of variances). For abnormally distributed variables, comparisons were done using the Kruskal-Wallis test, followed by Dunn’s post hoc test (with Bonferroni correction) if significant. Correlations were assessed using Spearman’s rank-order correlation.

Categorical variables were summarized as counts and frequencies. The associations were tested using Pearson’s Chi-square test for independence of observations (for nominal variables, replaced by Fisher’s exact test if the expected count was less than 5 in 20% or more of cells) or the Chi-squared Test for Trend in Proportions (for ordinal variables). An analysis of the ROC curve was performed to assess the discriminatory performance of the assessed measurements for predicting the need for MV and mortality. Optimal cut-off points were calculated for each measurement, along with sensitivity, specificity, overall accuracy, and predictive values. Survival analysis was performed to assess the impact of the studied parameters on time to death. Kaplan-Meier curves were constructed, and the Log-rank test was performed. A *p*-value < 0.05 was selected to interpret the results of statistical tests.

## Results

The current study enrolled 145 acutely ALP-poisoned patients who required ICU admission, as illustrated in Fig. 1. Table 1 revealed that the median age of the included patients was 19 years (IQR: 17–28 years). The majority of them were females (58.6% versus 41.1% of males). There were no significant age or sex variations regarding the quartiles of CVP measurements. All the studied patients had been poisoned by ingestion with a suicidal intent, with a median delay time of two hours. Almost (84.1%) of the studied patients were conscious with normal pupils (86.2%) and chest examination (95.2%). Vomiting represented the most common gastrointestinal manifestation (62.1%) versus epigastric tenderness and abdominal pain (22.1% and 12.4%, respectively). There were no significant differences between the route, mode of poisoning, delay time, the clinical manifestations on one side, and the quartiles of CVP measurements on the other side. On the other hand, agitation was detected in about 23.4% of the studied patients, and was significantly reported in patients with higher CVP measurements, especially Q4 (32.4%, *p* = 0.047).

**Table 1:** Demographic characteristic, history of toxic exposure and clinical manifestations of the studied patients stratified by quartiles of mean CVP level

Characteristic	All patients	CVP quartiles				
	Overall *n* = 145 (100%)	Q1 *n* = 40 (28%)	Q2 *n* = 33 (23%)	Q3 *n* = 47 (32%)	Q4 *n* = 25 (17%)	*P*-value
Age (years), Median [IQR] (Range)	19 [17–28] (14–57)	18 [17–22] (15–40)	19 [16–24] (15–53)	21 [18–36] (14–57)	23 [16–30] (14–53)	0.087*a*
Sex, n (%)						0.081*b*
Male	60 (41.4%)	15 (25%)	9 (15%)	21 (35%)	15 (25%)	
Female	85 (58.6%)	25 (29.4%)	24 (28.2%)	26 (30.6%)	10 (11.8%)	
Amount (tablet), Median [IQR] (Range)	1 [0.5–1] (0.3–3)	1 [0.5–1] (0.3–2)	1 [0.5–1] (0.3–2)	1 [1–1] (0.3–3)	1 [1–1] (0.3–2)	0.092*a*
Route, n (%)						> 0.999*c*
Ingestion	145 (100%)	40 (27.6%)	33 (22.8%)	47 (32.4%)	25 (17.2%)	
Inhalation	0 (0%)	0 (0%)	0 (0%)	0 (0%)	0 (0%)	
Mode, n (%)						> 0.999*c*
Suicidal	145 (100%)	40 (27.6%)	33 (22.8%)	47 (32.4%)	25 (17.2%)	
Accidental	0 (0%)	0 (0%)	0 (0%)	0 (0%)	0 (0%)	
Delay (hour), median [IQR] (Range)	2 [1–3.5] (0.3–34)	2 [1–3.5] (0.5–5)	2.5 [1.5–3] (0.5–6)	2 [1–4] (0.3–8)	2 [1–3.5] (0.5–34)	0.783*a*
Consciousness level, n (%)						0.178*c*
Conscious	122 (84.1%)	37 (30.3%)	28 (23%)	39 (32%)	18 (14.8%)	
Disturbed	23 (15.9%)	3 (13%)	5 (21.7%)	8 (34.8%)	7 (30.4%)	
Agitation, n (%)	34 (23.4%)	6 (17.6%)	8 (23.5%)	9 (26.5%)	11 (32.4%)$+	0.047* *b*
Vomiting, n (%)	90 (62.1%)	29 (32.2%)	20 (22.2%)	29 (32.2%)	12 (13.3%)	0.263*b*
Epigastric tenderness, n (%)	32 (22.1%)	12 (37.5%)	6 (18.8%)	9 (28.1%)	5 (15.6%)	0.562*b*
Abdominal pain, n (%)	18 (12.4%)	6 (33.3%)	4 (22.2%)	6 (33.3%)	2 (11.1%)	0.870*c*
Pupil, n (%)						0.360*c*
Normal	125 (86.2%)	37 (29.6%)	29 (23.2%)	40 (32%)	19 (15.2%)	
Miosis	15 (10.3%)	3 (20%)	2 (13.3%)	6 (40%)	4 (26.7%)	
Dilated	5 (3.4%)	0 (0%)	2 (40%)	1 (20%)	2 (40%)	
Chest examination, n (%)						0.824*c*
Normal	138 (95.2%)	38 (27.5%)	32 (23.2%)	44 (31.9%)	24 (17.4%)	
Crepitations	3 (2.1%)	1 (33.3%)	0 (0%)	2 (66.7%)	0 (0%)	
Crepitations & wheeze	1 (0.7%)	0 (0%)	1 (100%)	0 (0%)	0 (0%)	
Diminished air entry	3 (2.1%)	1 (33.3%)	0 (0%)	1 (33.3%)	1 (33.3%)	

*CVP *central venous pressure; *Q* quartile; *IQR* interquartile range (25th − 75th percentiles); *n* number; a Kruskal-Wallis rank sum test; b Pearson’s Chi-squared test; *c* Fisher’s exact test; $-: Significantly lower probability than expected under the null hypothesis on examining adjusted residuals with Bonferroni correction $+: Significantly higher probability than expected under the null hypothesis on examining adjusted residuals with Bonferroni correction; * Significant at *p* < 0.05

Table 2 demonstrated that patients with high CVP measurements showed significantly lower GCS, O_2_ saturations, systolic, diastolic, mean arterial blood pressures, and MAP/CVP ratios, but higher respiratory rates, shock, and modified shock indices (*p*-values > 0.05). Evaluating patients on admission showed that those exhibiting higher CVP measurements had significantly lower pH, serum bicarbonate, potassium, and higher levels of RBS, G/K ratio, serum creatinine, AST, and total leucocyte count (*p*-values > 0.05), as illustrated in Table 3.

**Table 2:** Vital signs of the studied patients stratified by quartiles of mean CVP level

Characteristic		All patients	CVP quartiles				
		Overall *n* = 145 (100%)	Q1 *n* = 40 (28%)	Q2 *n* = 33 (23%)	Q3 *n* = 47 (32%)	Q4 *n* = 25 (17%)	*P*-value
GCS	Mean ± SD (Range)	14.3 ± 2.4 (3–15)	14.9 ± 0.3 (14–15)	14.5 ± 1.6 (7–15)	14.1 ± 2.6 (3–15)	13.2 ± 4 (3–15)	0.017* *a*
SBP (mmHg)	Mean ± SD (Range)	66.6 ± 23.4 (40–130)	80.3 ± 23.3 (40–130)	69.4 ± 21.6 (40–110)	63.2 ± 21.4 (40–120)	47.6 ± 13.9 (40–80)	< 0.001*a
DBP (mmHg)	Mean ± SD (Range)	37.7 ± 16.4 (20–90)	47.5 ± 16.6 (20–90)	37.9 ± 15.6 (20–80)	36 ± 14.8 (20–80)	24.8 ± 9.2 (20–50)	< 0.001* *a*
Pulse (beat/min)	Mean ± SD (Range)	93.4 ± 21.9 (40–159)	92.3 ± 17.8 (56–127)	91 ± 20.6 (40–135)	96.4 ± 22.1 (50–159)	92.6 ± 29 (40–148)	0.688*a*
MAP (mmHg)	Mean ± SD (Range)	47.3 ± 18.4 (26.7–103.3)	58.4 ± 18.3 (26.7–103.3)	48.4 ± 17.1 (26.7–90)	45 ± 16.7 (26.7–93.3)	32.4 ± 10.6 (26.7–60)	< 0.001* *a*
Shock index	Mean ± SD (Range)	1.6 ± 0.7 (0.6–4)	1.3 ± 0.5 (0.7–2.8)	1.4 ± 0.5 (0.7–2.9)	1.7 ± 0.8 (0.6–4)	2.1 ± 0.8 (1–3.7)	< 0.001* *a*
Modified shock index	Mean ± SD (Range)	2.3 ± 1.1 (0.9–6)	1.8 ± 0.8 (1–4.2)	2.1 ± 0.8 (1.1–4.3)	2.5 ± 1.2 (0.9–6)	3.1 ± 1.3 (1.4–5.6)	< 0.001* *a*
Respiratory rate (cycle/min)	Mean ± SD (Range)	25.8 ± 7.1 (7–48)	24 ± 5.4 (16–36)	24.2 ± 5 (16–35)	28.4 ± 6.8 (16–48)	25.8 ± 10.8 (7–48)	0.006* *a*
Temperature (°C)	Mean ± SD (Range)	36.7 ± 0.3 (35.9–37.5)	36.8 ± 0.3 (35.9–37.5)	36.8 ± 0.3 (36.3–37.2)	36.7 ± 0.3 (36–37.5)	36.6 ± 0.4 (36–37.2)	0.109*a*
O_2_ saturation (%)	Mean ± SD (Range)	86.4 ± 11.7 (46–100)	91.6 ± 8.6 (51–100)	87.8 ± 9.9 (65–98)	83.6 ± 13.3 (46–100)	81.5 ± 11.7 (50–100)	< 0.001* *a*
MAP/CVP ratio	Median [IQR] (Range)	2 [1.16–3.06] (0.78–20.67)	3.92 [3–5.88] (1.57–20.67)	2.32 [1.4–2.81] (1.16–4.5)	1.79 [1.07–2.13] (0.95–3.59)	0.89 [0.89–0.97] (0.78–2)	< 0.001* *b*

*CVP* central venous pressure; *Q* quartile; *GCS* glasgow coma scale; *SBP *systolic blood pressure; *DBP* diastolic blood pressure; *MAP *mean arterial pressure; *O*_*2*_ oxygen; *IQR* interquartile range (25th − 75th percentiles); *n* number; *SD* standard deviation; *a* One-way analysis of means (not assuming equal variances); *b* Kruskal-Wallis rank sum test; * Significant at *p* < 0.05

**Table 3:** Laboratory investigations of the studied patients stratified by quartiles of mean CVP level

Characteristic		All patients	CVP quartiles				
		Overall *n* = 145 (100%)	Q1 *n* = 40 (28%)	Q2 *n* = 33 (23%)	Q3 *n* = 47 (32%)	Q4 *n* = 25 (17%)	*P*-value
pH	Mean ± SD (Range)	7.31 ± 0.14 (6.64–7.6)	7.36 ± 0.1 (7.04–7.6)	7.33 ± 0.1 (7.11–7.46)	7.29 ± 0.14 (6.69–7.53)	7.25 ± 0.17 (6.64–7.57)	0.003* *c*
HCO_3_ (mEq/L)	Mean ± SD (Range)	13.3 ± 4.6 (2.6–26.8)	14.1 ± 4.4 (5–23.3)	14.8 ± 3.7 (6–20.8)	13 ± 4.7 (2.6–26.8)	10.5 ± 4.5 (3.9–19.8)	0.002* *c*
PaCO_2_ (mmHg)	Mean ± SD (Range)	25.8 ± 9.3 (10–71.1)	23.6 ± 6.2 (10.6–38.1)	28 ± 8.2 (13.4–47.7)	27.1 ± 10.1 (12.5–53.8)	23.9 ± 12.2 (10–71.1)	0.107*c*
RBS (mg/dl)	Median [IQR] (Range)	132 [112–171] (35–442)	122.5 [103.5–143.5] (35–348)	132 [123–171] (49–351)	131 [114–185] (42–442)	161 [133–207] (72–441)	0.021* *b*
Na (mmol/L)	Mean ± SD (Range)	142.2 ± 6.4 (120–175)	141.5 ± 4.9 (128.5–150)	143.1 ± 5.7 (130.2–154.9)	142.6 ± 6.1 (131.4–159.8)	141.5 ± 9.4 (120–175)	0.650*c*
K (mmol/L)	Mean ± SD (Range)	3.61 ± 0.54 (1.11–5.3)	3.83 ± 0.46 (2.9–5.3)	3.52 ± 0.48 (2.7–4.8)	3.61 ± 0.60 (1.11–4.7)	3.39 ± 0.52 (2.2–4)	0.008* *c*
Glucose/K ratio	Median [IQR] (Range)	2.09 [1.63–2.86] (0.57–13.1)	1.82 [1.4–2.25] (0.57–5.22)	2.2 [1.9–2.99] (0.88–6.09)	2.06 [1.63–2.96] (0.63–13.1)	2.84 [2.13–3.72] (1.11–6.28)	0.001* *b*
Mg (mmol/L)	Mean ± SD (Range)	2.09 ± 0.38 (1.14–3.7)	2.16 ± 0.35 (1.5–2.99)	2.02 ± 0.36 (1.14–3.1)	2.07 ± 0.45 (1.35–3.7)	2.11 ± 0.28 (1.5–2.6)	0.415*c*
Urea (mg/dL)	Mean ± SD (Range)	31.8 ± 9.4 (14–78)	32.1 ± 12.6 (14–78)	29.6 ± 7.4 (15–49)	32.6 ± 6.6 (16–46)	32.5 ± 10.3 (20–60)	0.529*c*
Creatinine (mg/dL)	Mean ± SD (Range)	1.07 ± 0.27 (0.5–1.9)	0.98 ± 0.26 (0.5–1.7)	1.02 ± 0.18 (0.5–1.3)	1.13 ± 0.31 (0.78–1.9)	1.17 ± 0.27 (0.7–1.7)	0.007* *c*
AST (U/L)	Median [IQR] (Range)	24.1 [16–31] (8–666)	27 [18–34.5] (10–238)	23 [15–29] (10–79)	19 [14–31] (8–89)	29 [21–33] (13–666)	0.020* *b*
ALT (U/L)	Median [IQR] (Range)	22 [14–32] (7–543)	20.5 [14.5–29] (7–180)	19 [13–32] (10–85)	22 [13–31.5] (7–91)	28 [15.8–36] (1–543)	0.256*b*
Hb (g/dL)	Mean ± SD (Range)	11.7 ± 1.7 (7.8–18)	11.7 ± 2.2 (7.8–18)	11.5 ± 1.7 (7.8–15.6)	11.8 ± 1.3 (9–14.6)	11.8 ± 1.4 (9.2–15)	0.921*a*
Platelet count (×10^3^ /L)	Mean ± SD (Range)	237.3 ± 67.8 (38–440)	237.9 ± 77.4 (38–390)	240.7 ± 62.1 (152–432)	230.9 ± 52.5 (140–372)	243.9 ± 85.4 (88–440)	0.837*a*
Leucocytic count (x10^3^ /L)	Mean ± SD (Range)	9.7 ± 3.7 (1.8–23.7)	8.4 ± 3.2 (1.8–15.1)	9.9 ± 2.9 (5.1–15.2)	10.2 ± 4.3 (3.6–23.7)	10.8 ± 3.3 (4.9–17)	0.040* *c*

*CVP* central venous pressure; *Q* quartile; *MAP* mean arterial pressure; *HCO*_*3*_ bicarbonate; *PaCO*_*2*_ partial arterial carbon dioxide pressure; *RBS *random blood sugar; *Na* sodium; *K* potassium; *Mg* magnesium; *AST* aspartate aminotransferase, *ALT* alanine aminotransferase; *Hb* hemoglobin; *IQR* interquartile range (25th − 75th percentiles); *n* number; *SD* standard deviation; *a* One-way analysis of means (not assuming equal variances); *b* Kruskal-Wallis rank sum test; *c* One-way analysis of means; * Significant at *p* < 0.05

Moreover, Table 4 showed that there was no statistical difference between the quartiles of CVP measurements when examining the ECG of the studied patients. Regarding abnormal ECG findings, atrial fibrillation was significantly detected in patients with high CVP measurements, while supraventricular tachycardia and ST segment changes were significantly reported in patients with low CVP measurements (*p*-values < 0.05). Based on the reported PSS on admission, severe cases significantly represented 59.6% and 76% of the studied patients belonged to Q3 and Q4 groups of CVP levels, respectively. Studying the outcomes of the included patients revealed that in-hospital mortality and need for MV were significantly predominant in Q3 and Q4 groups of CVP levels (89.4% and 92% & 91.5% and 96%, respectively) (*p-values* < 0.001). Moreover, patients with high CVP measurements had significantly lower total vasopressor doses and shorter hospital stays (*p-*values < 0.001). In addition, post hoc tests of different variables of the studied patients stratified by quartiles of mean CVP level were illustrated in Supplementary Table 1.

**Table 4:** ECG changes and outcomes of the studied patients stratified by quartiles of mean CVP level

Characteristic		All patients	CVP quartiles				
		Overall *n* = 145 (100%)	Q1 *n* = 40 (28%)	Q2 *n* = 33 (23%)	Q3 *n* = 47 (32%)	Q4 *n* = 25 (17%)	*P*-value
ECG abnormality	n (%)						0.344*a*
Abnormal		96 (66.2%)	27 (67.5%)	23 (69.7%)	33 (70.2%)	13 (52%)	
Normal		49 (33.8%)	13 (32.5%)	10 (30.3%)	14 (29.8%)	12 (48%)	
Prolonged QTc interval	n (%)	37 (25.5%)	11 (27.5%)	8 (24.2%)	13 (27.7%)	5 (20%)	0.649*a*
Ventricular tachycardia/ fibrillation	n (%)	4 (2.8%)	0 (0%)	3 (9.1%)	0 (0%)	1 (4%)	0.839*a*
T-wave changes	n (%)	7 (4.8%)	2 (5%)	4 (12.1%)	1 (2.1%)	0 (0%)	0.172*a*
Atrial fibrillation	n (%)	34 (23.4%)	6 (15%)	4 (12.1%)	17 (36.2%)	7 (28%)	0.032* *a*
Supraventricular tachycardia	n (%)	7 (4.8%)	4 (10%)	2 (6.1%)	1 (2.1%)	0 (0%)	0.037* *a*
Heart block	n (%)	6 (4.1%)	2 (5%)	1 (3%)	1 (2.1%)	2 (8%)	0.802*a*
ST segment elevation/ depression	n (%)	9 (6.2%)	5 (12.5%)	3 (9.1%)	0 (0%)	1 (4%)	0.035* *a*
PSS	n (%)						< 0.001* *a*
Mild		5 (3.4%)	2 (5%)	2 (6.1%)	1 (2.1%)	0 (0%)	
Moderate		71 (49%)	31 (77.5%)	16 (48.5%)	18 (38.3%)	6 (24%)	
Severe		69 (47.6%)	7 (17.5%)	15 (45.5%)	28 (59.6%)	19 (76%)	
Length of hospital stay (hours)	Median [IQR] (Range)	9 [5–22.5] (1–173)	36.5 [11–74] (3.5–173)	12 [7–27] (2.5–120)	6 [4–9.5] (1–35)	5.5 [3.5–6.5] (2–84)	< 0.001* *b*
Mortality	n (%)						< 0.001* *a*
Survived		38 (26.2%)	21 (52.5%)	10 (30.3%)	5 (10.6%)	2 (8%)	
Dead		107 (73.8%)	19 (47.5%)	23 (69.7%)	42 (89.4%)	23 (92%)	
Need for mechanical ventilation	n (%)						< 0.001* *a*
Yes		104 (71.7%)	17 (42.5%)	20 (60.6%)	43 (91.5%)	24 (96%)	
No		41 (28.3%)	23 (57.5%)	13 (39.4%)	4 (8.5%)	1 (4%)	
Total dose of vasopressor (mg)	Median [IQR] (Range)	15.4 [7.4–33.2] (0.6–266)	30.9 [13.8–43.7] (1.7–266)	16 [7–43.1] (1.9–216.6)	12.3 [7.4–28] (0.6–101.6)	11.1 [6.5–18.5] (2.5–133)	< 0.001* *b*

*CVP* central venous pressure; *Q* quartile; *ECG* electrocardiography; *QTc* corrected QT; *PSS* poisoning Severity Score; *IQR* interquartile range (25th−75th percentiles); *n* number; *a* Chi-squared Test for Trend in Proportions; b Kruskal-Wallis rank sum test; * Significant at *p* < 0.05

The measurement of CVP level after the administration of vasopressors could be reported in only 50 patients out of all studied patients. The mean of reported CVP levels was 17.6 mmHg (SD ± 8.3 mmHg), and ranged from 4 to 33 mmHg. Among these patients, 26 (52%) were survivors, compared with 24 non-survivors (48%). The analysis of the change in CVP level after the administration of vasopressors, based on the Welch Two Sample t-test, detected that survivors demonstrated a significant mean reduction in CVP measurements of − 4.5 mmHg (SD ± 7.2 mmHg), ranging from − 19 to 8 mmHg. In contrast, non-survivors had a mean increase of + 0.6 mmHg (SD ± 6.4 mmHg), and ranged from − 11 to 14 mmHg (*p* = 0.011).

Inspecting Table 5 reveals a significant negative association between the CVP measurement and GCS, SBP, DBP, MAP, O_2_ saturation, pH, HCO_3_, and potassium levels. Furthermore, there was a significant positive correlation between CVP and the reported PSS of the studied patients on admission (*r* = 0.399, *p* < 0.001). On the other hand, we observed significant negative correlations between the CVP and both the length of hospital stay and total dose of vasopressors taken (*r* = −0.521 and − 0.337, respectively; *p-values* < 0.001). Thus, acute ALP-poisoned patients with higher CVP measurements rapidly deteriorated and were discharged from the hospital with a short hospital stay.

**Table 5:** Spearman correlation analysis between CVP from one side and the different variables of acute ALP-poisoned patients from the other side

Variables		rho^a^	*P*-value
CVP (mmHg)	Age (years)	0.141	0.092
CVP (mmHg)	Delay (hour)	0.015	0.856
CVP (mmHg)	Amount (tablet)	0.209	0.012*
CVP (mmHg)	GCS	− 0.204	0.014*
CVP (mmHg)	SBP (mmHg)	− 0.460	< 0.001*
CVP (mmHg)	DBP (mmHg)	− 0.449	< 0.001*
CVP (mmHg)	Pulse (beat/min)	− 0.059	0.481
CVP (mmHg)	MAP	− 0.463	< 0.001*
CVP (mmHg)	Shock index	0.376	< 0.001*
CVP (mmHg)	Modified shock index	0.392	< 0.001*
CVP (mmHg)	Respiration (cycle/min)	0.206	0.013*
CVP (mmHg)	Temperature (°C)	− 0.219	0.008*
CVP (mmHg)	RBS (mg/dl)	0.250	0.002*
CVP (mmHg)	O₂ saturation (%)	− 0.390	< 0.001*
CVP (mmHg)	pH	− 0.371	< 0.001*
CVP (mmHg)	HCO₃ (mEq/L)	− 0.276	0.001*
CVP (mmHg)	PaCO₂ (mmHg)	− 0.025	0.762
CVP (mmHg)	Na (mmol/L)	− 0.015	0.855
CVP (mmHg)	K (mmol/L)	− 0.207	0.013*
CVP (mmHg)	Glucose/K ratio	0.302	< 0.001*
CVP (mmHg)	Mg (mmol/L)	− 0.017	0.843
CVP (mmHg)	Urea (mg/dL)	0.123	0.141
CVP (mmHg)	Creatinine (mg/dL)	0.227	0.006*
CVP (mmHg)	AST (U/L)	0.040	0.634
CVP (mmHg)	ALT (U/L)	0.157	0.059
CVP (mmHg)	Hb (g/dL)	0.029	0.726
CVP (mmHg)	Platelet count (×10⁹/L)	− 0.033	0.695
CVP (mmHg)	Leucocytic count (x10⁹/L)	0.236	0.004*
CVP (mmHg)	Length of hospital stay (hours)	− 0.521	< 0.001*
CVP (mmHg)	Total dose of vasopressor (mg)	− 0.337	< 0.001*
CVP (mmHg)	PSS	0.399	< 0.001*

*CVP* central venous pressure; *GCS* glasgow coma scale; *SBP* systolic blood pressure; *DBP* diastolic blood pressure; *MAP* mean arterial pressure; *O*_*2*_ oxygen; *HCO*_*3*_ bicarbonate; *PaCO*_*2*_ partial arterial carbon dioxide pressure; *RBS* random blood sugar; *Na* sodium; *K* potassium; *Mg* magnesium; *AST* aspartate aminotransferase, *ALT* alanine aminotransferase; *PSS* poisoning severity score

^a^Coefficient of Spearman’s rank-order correlation; * Significant at *p* < 0.05

**Fig. 2:**
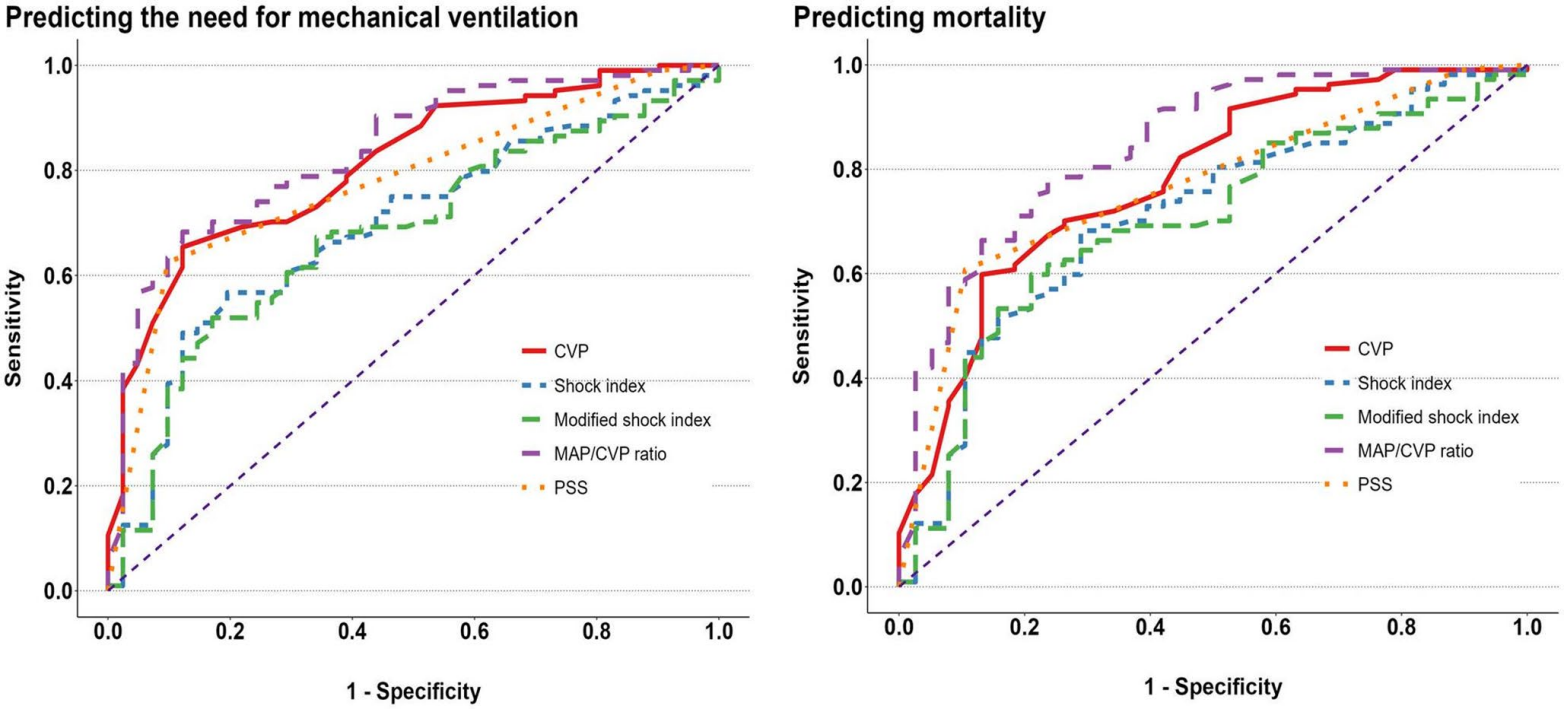
Receiver operating characteristic curves of the studied parameters as predictors of the need for mechanical ventilation and mortality in acute ALP-poisoned patients admitted to the ICU

The results of ROC analyses assessing the predictors of the need for MV in acute ALP-poisoned patients admitted to the ICU are presented in Table 6 and Fig. 2. The CVP measurement at a cut-off higher than 23.5 mmHg was a significant predictor of the need for MV in acutely ALP-poisoned patients with an AUC of 0.812, a sensitivity of 65.4%, a specificity of 87.8%, and, an overall accuracy 71.7%. Additionally, a MAP/CVP ratio less than 2.03 was a significant predictor of the need for MV, exhibiting an AUC of 0.836 and an overall accuracy of 73.8%. It correctly classified 68.3% of patients who needed MV and excluded 87.7% of patients who did not need MV. In addition, there were no significant differences between the CVP measurement, MAP/CVP ratio, and PSS in pairwise comparisons of the AUCs for predicting the need for MV. On the other hand, CVP and MAP/CVP ratio had better performance than SI and modified SI in predicting the need for MV in ALP-poisoned patients (*p* < 0.05).

**Table 6:** Receiver-operating characteristics curve analyses to assess the performance of CVP and MAP/CVP ratio in the prediction of need for mechanical ventilation of acute ALP-poisoned patients

Outcomes	Predictors	AUC (95% CI)	Cut-off	Sensitivity (%)	Specificity (%)	PPV (%)	NPV (%)	Accuracy (%)
Need for mechanical ventilation	CVP	0.812 (0.735 to 0.880)	≥ 23.5	65.4	87.8	93.2	50.0	71.7
	MAP/CVP ratio	0.836 (0.756 to 0.907)	≤ 2.03	68.3	87.8	93.4	52.2	73.8
	Shock index	0.692 (0.597 to 0.783)	≥ 1.44	56.7	80.5	88.1	42.3	63.4
	Modified shock index	0.678 (0.587 to 0.771)	≥ 2.14	51.9	82.9	88.5	40.5	60.7
	PSS	0.778 (0.708 to 0.842)	≥ 3	62.5	90.2	94.2	48.7	70.3
Comparison		***P*** -value	Method					
Shock index vs. CVP		0.020*	DeLong’s test for two correlated ROC curves					
Shock index vs. MAP/CVP ratio		0.001*	Bootstrap test for two correlated ROC curves					
Shock index vs. Modified shock index		0.191	DeLong’s test for two correlated ROC curves					
Shock index vs. PSS		0.034*	DeLong’s test for two correlated ROC curves					
CVP vs. MAP/CVP ratio		0.351	Bootstrap test for two correlated ROC curves					
CVP vs. Modified shock index		0.009*	DeLong’s test for two correlated ROC curves					
CVP vs. PSS		0.483	DeLong’s test for two correlated ROC curves					
MAP/CVP ratio vs. Modified shock index		< 0.001*	Bootstrap test for two correlated ROC curves					
MAP/CVP ratio vs. PSS		0.100	Bootstrap test for two correlated ROC curves					
Modified shock index vs. PSS		0.015*	DeLong’s test for two correlated ROC curves					

*AUC* area under the curve; *CI* confidence interval; *NPV* negative predictive value; *PPV* positive predictive value; *ROC* receiver operating characteristics; *CVP* central venous pressure; *MAP* mean arterial pressure; *PSS* poisoning severity score; * Significant at *p* < 0.05

Table 7 and Fig. 2 depicted that, at a cut-off of more than 25 mmHg, the CVP could be a significant predictor of mortality in acute ALP-poisoned patients admitted to the ICU, showing an AUC of 0.778, a sensitivity of 62.5%, and a specificity of 90.2%. Similarly, a MAP/CVP ratio less than 2.48 was another significant predictor of mortality, with an AUC of 0.846 and an overall accuracy of 77.2%. It could correctly classified 77.6% of deaths and correctly excluded 76.3% of survived patients. Furthermore, it was noticed that the MAP/CVP ratio had a better performance than CVP measurement, SI, and modified SI in predicting mortality in ALP-poisoned patients (*p-values* = 0.042, < 0.001, and < 0.001, respectively).

**Table 7:** Receiver-operating characteristics curve analyses to assess the performance of CVP and MAP/CVP ratio in the prediction of mortality of acute ALP-poisoned patients

Outcomes	Predictors	AUC (95% CI)	Cut-off	Sensitivity (%)	Specificity (%)	PPV (%)	NPV (%)	Accuracy (%)
Mortality	CVP	0.782 (0.697 to 0.863)	≥ 25	59.8	86.8	92.8	43.4	66.9
	MAP/CVP ratio	0.846 (0.767 to 0.915)	≤ 2.48	77.6	76.3	90.2	54.7	77.2
	Shock index	0.711 (0.613 to 0.799)	≥ 1.27	68.2	71.1	86.9	44.3	69
	Modified shock index	0.699 (0.607 to 0.792)	≥ 1.99	59.8	78.9	88.9	41.1	64.8
	PSS	0.768 (0.697 to 0.830)	≥ 3	60.7	89.5	94.2	44.7	68.3
Comparison		***P*** -value	Method					
Shock index vs. CVP		0.211	DeLong’s test for two correlated ROC curves					
Shock index vs. MAP/CVP ratio		< 0.001*	Bootstrap test for two correlated ROC curves					
Shock index vs. Modified shock index		0.329	DeLong’s test for two correlated ROC curves					
Shock index vs. PSS		0.164	DeLong’s test for two correlated ROC curves					
CVP vs. MAP/CVP ratio		0.042*	Bootstrap test for two correlated ROC curves					
CVP vs. Modified shock index		0.146	DeLong’s test for two correlated ROC curves					
CVP vs. PSS		0.791	DeLong’s test for two correlated ROC curves					
MAP/CVP ratio vs. Modified shock index		< 0.001*	Bootstrap test for two correlated ROC curves					
MAP/CVP ratio vs. PSS		0.057	Bootstrap test for two correlated ROC curves					
Modified shock index vs. PSS		0.099	DeLong’s test for two correlated ROC curves					

**Fig. 3:**
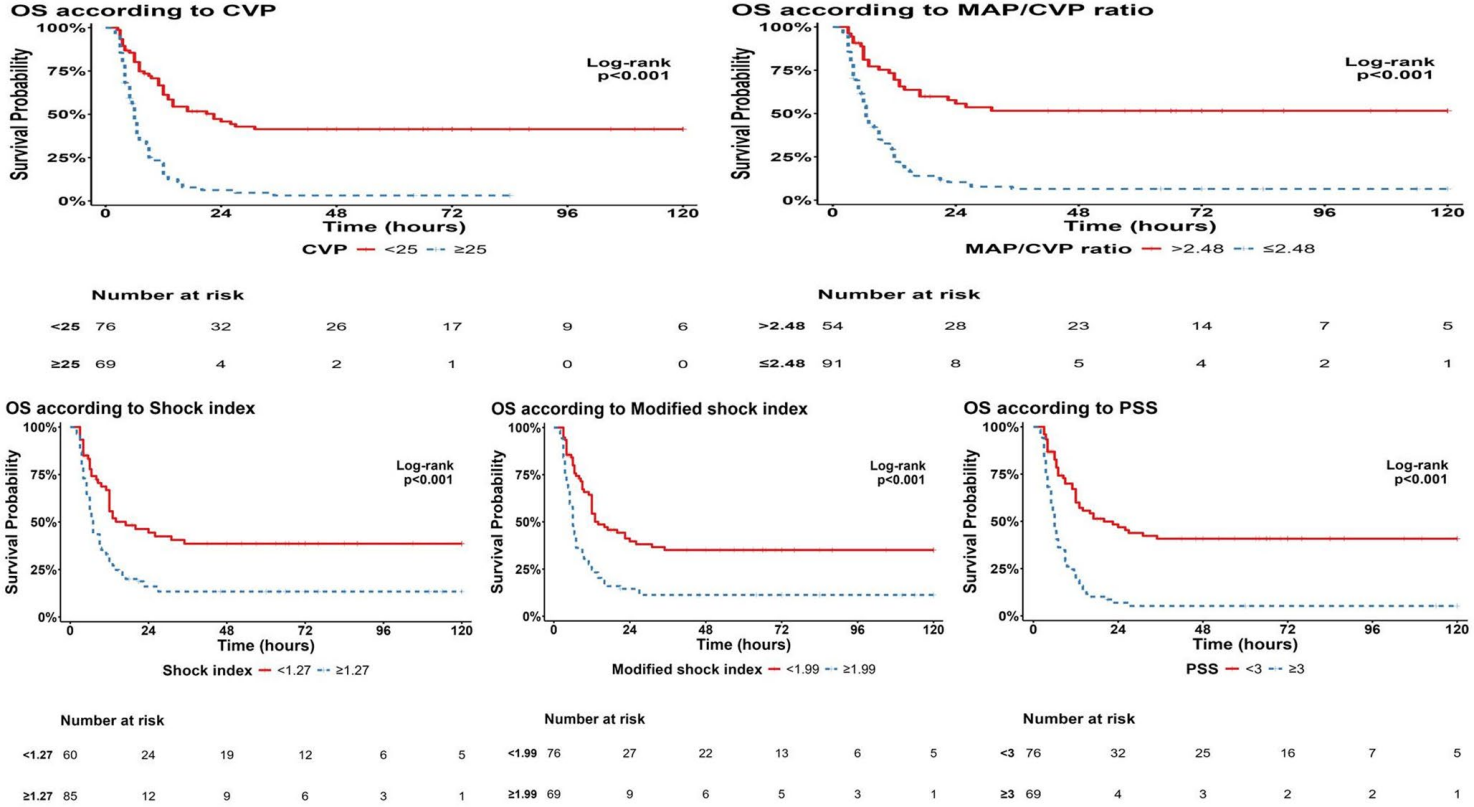
Kaplan-Meier analysis of the survival probability of acute ALP-poisoned patients stratified by the studied parameters cutoff level

The studied patients demonstrated a significant decrease in survival probability and time to death or discharge in patients with CVP measurement ≥ 25 mmHg compared to those with CVP *<* 25 mmHg (6 versus 22.5 h; *p <* 0.001), and in patients with MAP/CVP ratio lower than 2.48 (6.5 h, *p <* 0.001). Moreover, patients with SI and modified SI values above 1.27 and 1.99, respectively, had significantly shorter mean survival times (*p-*values *<* 0.001). Additionally, patients with PSS < 3 had a significantly higher mean survival time of 20 h compared to 6 h for those with PSS ≥ 3 (*p <* 0.001), as shown in Table 8 and Fig. 3.

**Table 8:** Overall survival according to the studied parameters in acute ALP-poisoned patients

Characteristic	Patients (*n*)	Events (*n*)	Median OS in hours (95% CI)	*P*-value* a*
CVP				< 0.001*
<25 mmHg	76	43	22.5 (12, 33)	
≥25mmHg	69	64	6 (5.4, 6.6)	
MAP/CVP ratio				< 0.001*
>2.48	54	25	NA	
≤2.48	91	82	6.5 (6, 9)	
Shock index				< 0.001*
<1.27	60	34	17 (3, 31)	
≥1.27	85	73	7 (6.1, 7.9)	
Modified shock index				< 0.001*
<1.99	76	46	14 (12, 31)	
≥1.99	69	61	6 (5, 7)	
PSS				< 0.001*
<3	76	42	20 (7.2, 32.8)	
≥3	69	65	6 (5.4, 6.6)	

*n* number; *OS* overall survival; *CVP* central venous pressure; *MAP* mean arterial pressure; *PSS* poisoning severity score; *a* Log-rank test; * Significant at *p*<0.05

## Discussion

Numerous studies have shown that ALP was the most common poison to cause cardiotoxicity and adverse cardiovascular events, including shock, ventricular arrhythmias, and death [13–15, 41, 53]. To our knowledge, it is yet unknown how CVP measurements are helpful for the management and follow-up of ICU-admitted patients who are suffering from acute ALP poisoning. Thus, the current study aimed to evaluate the predictive application of the novel parameters CVP and MAP/CVP ratio in the assessment of poisoning severity and the poor outcomes in acute ALP-poisoned patients who were admitted to the ICU.

Analysis of the poor outcomes among the included patients revealed that 71.7% required MV. Adel et al. [41] and Khalifa et al. [54] were in the same line. Nevertheless, Lashin et al. [55] found ALP as the most common poison that mandated MV among their studied intoxicated patients. This could be referred to hypoxia, acute respiratory distress syndrome, and pulmonary edema, which are commonly induced by acute ALP poisoning [41]. In addition, our study found that MV need rates were significantly higher in patients with high CVP measurements, and these patients also showed lower O_2_ saturations and much higher respiratory rates. These findings aligned with those of Abd Elghany et al. [56] and Sheta et al. [57]. This could be referred to as raised CVP leading to increased capillary hydrostatic pressure, promoting pulmonary edema that requires MV to maintain adequate gas exchange and relieve respiratory muscle fatigue [58]. Furthermore, elevated CVP frequently accompanies right heart failure, causing ventilation-perfusion mismatch and hypoxemia [59]. Additionally, patients with high CVP and poor outcomes had significantly low GCS grades that made them more vulnerable to aspiration pneumonia due to absent protective airway reflexes [60].

According to the current study, the mortality rate was 73.8%. Khalaf et al. [3], Dorooshi et al. [61], and Elmehy et al. [62] had reported similar mortality rates. The powerful toxic effects of acute ALP poisoning, increasingly worsening clinical manifestations, and lack of any specific antidote all contribute to these high mortality rates [9, 63]. This was followed by shorter hospital stays and lower overall vasopressor doses. Furthermore, a higher percentage of the studied patients with severe PSS may serve as an additional reason [54]. Moreover, the studied patients with high CVP measurements had a significantly higher rate of in-hospital mortality. Similarly, previous literature found that increased CVP levels are associated with worsened outcomes and in-hospital mortality in patients with critical medical conditions [42, 64].

In the current study, patients with high CVP and poor outcomes had significantly lower SBP, DBP, and MAP. These findings are similar to those of Ahmed et al. [65] and Pannu et al. [66]. One of the vital factors that leads to poor prognosis in cases of ALP poisoning is hypotension [66], which could be explained by the massive loss of intravascular fluid due to vascular wall insufficiency, myocardial injury, adrenal gland dysfunction, and the significant collapse of the circulatory system induced by the direct cardiotoxic impacts of phosphine gas [30, 67]. Hence, the severity of hypotension induced by ALP poisoning is considered the most significant predictor of ALP outcomes [30]. Therefore, monitoring of CVP measures is now recommended for guiding the fluid therapy in acute ALP-poisoned patients with cardiovascular instability [28].

Furthermore, our results confirmed that survivors demonstrated a significant mean reduction in CVP measurements (–4.5 mmHg), whereas non-survivors demonstrated a significant mean increase (+ 0.6 mmHg) in CVP measurements after vasopressor administration. Therefore, CVP should be kept as low as possible within the normal physiological limits in acute ALP-poisoned patients. Similarly, Su et al. [42] reported that lower CVP values are associated with increased cardiac output, which may improve 28-day mortality in critically ill patients with circulatory shock. Several mechanisms could explain why lower CVP measurements are crucial for maintaining normal physiological organ function and, hence, a higher survival rate [28]. Firstly, reduced venous return pressure can improve kidney congestion [68]. Second, elevated CVP may influence pulmonary circulation and oxygenation [69, 70]. Third, CVP can affect organ and tissue perfusion, including microcirculatory perfusion [71] and cerebral blood flow regulation [72].

Additionally, patients with high CVP and poor outcomes in the current study had significantly lower pH and HCO_3_. In alignment with these findings, Sagah and Elhawary [73] found that non-survivors had significantly lower pH and HCO_3_. Additionally, decreased serum HCO_3_ level below 18 mmol/L and poisoning with ALP were significantly associated with increased risk of all poor outcomes as reported by El-Sarnagawy et al. [74]. This could be referred to as metabolic acidosis, which is induced by cytochrome c oxidase inhibition and severe hypoperfusion, and is known to be a major cause of fatality in acute ALP poisoning [6, 75]. Regarding abnormal ECG findings, atrial fibrillation was significantly detected in the studied patients with high CVP measurements, who are more predisposed to poor outcomes. This finding was in accordance with Befin and Chandra [20], who found that the most common pathological ECG manifestation in acute ALP poisoning was atrial fibrillation with 100% mortality rate in those patients on the first day of admission. Furthermore, Hamidi et al. [76] reported that people with atrial fibrillation had significantly greater changes in CVP than those without. This could be explained by the fact that an elevated CVP reflects volume overload in the right atrium or ventricle, which stretches the atrial wall due to atrial distention and triggers atrial fibrillation [77].

According to our study, the MAP/CVP ratio shows good discriminatory power (AUCs = 0.836 and 0.846) for predicting the need for MV and in-hospital mortality, respectively, in acute ALP-poisoned patients. Similarly, Mohamedali et al. [40] reported that the MAP/CVP ratio < 7.5 had a high predictive value for early right ventricular failure and increased mortality risk after left ventricular assist device placement. They attributed this to the fact that MAP reflects both SBP and DBP and systemic vascular resistance, thereby influencing left ventricular end-diastolic pressure, whereas CVP reflects volume status and right ventricular function. Thus, the MAP/CVP ratio incorporates the functionality of both ventricles into a single parameter. Moreover, the MAP/CVP ratio overlaps CVP measurement in predicting the need for MV and in-hospital mortality in patients poisoned with ALP; the CVP measurement (AUCs = 0.812 and 0.782, respectively) comes in second, followed by the PSS (AUCs = 0.778 and 0.768, respectively).

In addition, this study showed that both the MAP/CVP ratio and CVP measurement have a better performance to predict the need for MV and in-hospital mortality among ALP-poisoned patients than SI and MSI, which were introduced recently as simple, rapid, and applicable risk stratification predictors for patients diagnosed with acute ALP poisoning [49]. This could be referred to as SI and MSI, which are mainly used to evaluate acute hypovolemia and circulatory failure and incorporate heart rate, SBP, and DBP. Hence, their values will be misleading as the blood pressure remains normal during the compensatory phase of shock [78]. On the other hand, changes in circulating volume are reflected rapidly in the CVP when blood volume decreases. So, CVP monitoring is more accurate than measuring blood pressure [76].

Furthermore, we observed that both the MAP/CVP ratio and CVP showed comparable predictive ability to PSS for poor outcomes in patients with ALP poisoning and were significantly positively associated with the reported PSS. However, the PSS score’s clinical value for early emergency decision-making is limited by the inclusion of multiple complex clinical and laboratory data [79]. From the perspective of the above results, the CVP and MAP/CVP ratio offer reliable, easy, and objective bedside predictors of the severity of acute ALP poisoning and the probability of its outcomes. They also provide valuable information on hemodynamics and cardiovascular conditions, making it a beneficial assessment tool.

This study demonstrated a value > 23.5 mmHg as the best cut-off of CVP to predict need for MV and a value > 25 mmHg to predict mortality. For the MAP/CVP ratio, the best cut-off to predict the need for MV was ≤ 2.03, and the best cut-off to predict mortality was ≤ 2.48. So, this study was the first to provide risk values for both parameters in acute ALP poisoning. Additionally, using Kaplan-Meier analysis, the authors examined the effect of CVP cut-off on survival duration in patients with acute ALP poisoning. Compared to patients whose CVP < 25 mmHg and whose MAP/CVP ratio > 2.48, who had longer survival durations, our study showed that patients with a CVP ≥ 25 mmHg and a MAP/CVP ratio ≤ 2.48 should be promptly sent to emergent treatments because they had a significantly lower median survival time (6 and 6.5 h, respectively). Our previous findings highlight the critical role of early CVP measurements in triaging the high-risk ALP-poisoned patient for comprehensive, early supportive care.

The retrospective observational design is the main limitation of the current study. Hence, data on NT-proBNP, troponin T, or echocardiogram were not reported in patients’ medical records as they are not routinely done in TUPCC. In addition, previous ECGs before acute ALP poisoning were not available. Moreover, this study raised a dilemma for future research: whether elevated CVP represents a true prognostic factor or merely reflects advanced disease severity. The worsening clinical status of acute ALP-poisoned patients may lead to increased CVP primarily due to severe phosphine-induced myocardial depression. The released PH_3_ induces oxidative stress and lipid peroxidation, leading to acute toxic myocarditis and markedly reduced cardiac output [13, 80]. Moreover, the profound metabolic acidosis further depresses myocardial function and catecholamine responsiveness [54], while aggressive fluid resuscitation in shock may worsen venous congestion in the setting of pump failure [81]. Thus, the elevated CVP in acute ALP poisoning may indicate progressive cardiogenic shock and poor prognosis that necessitates rapid intervention to save the patient’s life.

## Recommendations

The current study recommends adopting the MAP/CVP ratio and CVP as early prognostic parameters in acutely ALP-poisoned patients. Lower MAP/CVP ratios and higher CVP measurements are alarming signs, warranting a higher risk of the need for MV and in-hospital mortality. Urgent referral to the ICU, initiating MV, and vasopressor therapy should be done without any delay. We further recommend validating the role of the MAP/CVP ratio and CVP as novel prognostic parameters in acute ALP poisoning in different poison control centers in Egypt and worldwide, allowing generalization of the reported findings.

## Conclusion

This study reported that high CVP measurements and lower MAP/CVP ratios are significant findings of acute ALP-poisoned patients associated with poor outcomes, including increased need for MV and in-hospital mortality. Moreover, early reductions in CVP within the normal physiological limits as possible during treatment of acute ALP-poisoned patients may result in a higher survival rate. Additionally, the value of continuous monitoring of CVP in acute ALP-poisoned patients admitted to the ICU was pointed out. This study highlighted the high discriminatory power of the MAP/CVP ratio for predicting the need for MV and in-hospital mortality in acute ALP-poisoned patients overlapping both CVP and PSS.

## Supplementary Information

Below is the link to the electronic supplementary material.

Supplementary Material 1

## Data Availability

Data are available upon reasonable request from the corresponding author.
