# Supplementary material for: Role of Central Venous Pressure and Mean Arterial Pressure to Central Venous Pressure Ratio in Assessing Poisoning Severity and Poor Outcomes in Intensive Care Unit-Admitted Acute Aluminum Phosphide-Poisoned Patients
Source: Cardiovasc Toxicol. 2026 Mar 14;26(3):34. doi: 10.1007/s12012-026-10103-0 (PMC12988964; doi:10.1007/s12012-026-10103-0)
Supplement: Supplementary file 1 — Supplementary Material 1 [file 12012_2026_10103_MOESM1_ESM.docx]

**Supplementary Table 1** Post hoc tests of different variables of the studied patients stratified by quartiles of mean CVP level

| **Variable** | **Q1 vs. Q2** | **Q1 vs. Q3** | **Q1 vs. Q4** | **Q2 vs. Q3** | **Q2 vs. Q4** | **Q3 vs. Q4** |
| --- | --- | --- | --- | --- | --- | --- |
| **GCS** | 0.393 | 0.183 | 0.155 | 0.873 | 0.417 | 0.702 |
| **SBP** | 0.176 | **0.004*** | **<0.001*** | 0.586 | **<0.001*** | **0.002*** |
| **DBP** | 0.061 | **0.006*** | **<0.001*** | 0.945 | **0.001*** | **0.001*** |
| **MAP** | 0.084 | **0.004*** | **<0.001*** | 0.820 | **<0.001*** | **0.001*** |
| **Shock index** | 0.535 | **0.007*** | **0.001*** | 0.171 | **0.007*** | 0.301 |
| **Modified Shock index** | 0.336 | **0.007*** | **<0.001*** | 0.309 | **0.007*** | 0.190 |
| **Respiratory rate** | 0.999 | **0.007*** | 0.868 | **0.011*** | 0.898 | 0.697 |
| **O_2_ saturation** | 0.323 | **0.007*** | **0.003*** | 0.379 | 0.147 | 0.897 |
| **CVP** | **<0.001*** | **<0.001*** | **<0.001*** | **<0.001*** | **<0.001*** | **<0.001*** |
| **MAP/CVP ratio** | **0.002*** | **<0.001*** | **<0.001*** | 0.096 | **<0.001*** | **0.002*** |
| **pH** | 0.735 | **0.042*** | **0.003*** | 0.461 | 0.065 | 0.548 |
| **PaCO₂** | 0.175 | 0.297 | 0.999 | 0.968 | 0.333 | 0.507 |
| **HCO₃** | 0.923 | 0.650 | **0.007*** | 0.301 | **0.002*** | 0.091 |
| **RBS** | 0.426 | 0.476 | **0.013*** | 1.000 | 1.000 | 0.619 |
| **Na** | 0.699 | 0.853 | 1.000 | 0.983 | 0.777 | 0.903 |
| **K** | 0.071 | 0.214 | **0.007*** | 0.896 | 0.760 | 0.330 |
| **Glucose/Potassium ratio** | 0.119 | 0.259 | **0.001*** | 1.000 | 0.559 | 0.145 |
| **Mg** | 0.379 | 0.654 | 0.960 | 0.942 | 0.779 | 0.960 |
| **Urea** | 0.693 | 0.994 | 0.999 | 0.511 | 0.674 | 1.000 |
| **Creatinine** | 0.928 | **0.034*** | **0.025*** | 0.205 | 0.127 | 0.945 |
| **ALT** | 1.000 | 1.000 | 0.470 | 1.000 | 0.465 | 0.589 |
| **AST** | 0.826 | 0.197 | 1.000 | 1.000 | 0.194 | **0.037*** |
| **Hb** | 0.988 | 0.998 | 0.996 | 0.919 | 0.926 | 1.000 |
| **Platelet count** | 0.998 | 0.962 | 0.992 | 0.882 | 0.999 | 0.899 |
| **Leucocytic count** | 0.300 | 0.100 | **0.048*** | 0.982 | 0.772 | 0.902 |
| **Length of hospital stay** | 0.192 | **<0.001*** | **<0.001*** | **0.019*** | **0.020*** | 1.000 |
| **Vasopressor dose** | 0.308 | **0.003*** | **0.001*** | 1.000 | 0.366 | 1.000 |

*P*-values from Games-Howell test for GCS, SBP, DBP, MAP, SI, Modified SI, RR, O_2_ saturation, CVP, Hb, and platelet count.

*P*-values from Tukey's HSD test for pH, PaCO_2_, HCO_3_, Na, K, Mg, urea, creatinine, and leucocytic count.

*P*-values from Dunn test with Bonferroni adjustment for MAP/CVP ratio, RBS, ALT, AST, Glucose/Potassium ratio, length of hospital stay and vasopressor dose.

* Significant at *p*<0.05.

CVP: Central venous pressure; Q: Quartile; GCS: Glasgow coma scale; SBP: Systolic blood pressure; DBP: Diastolic blood pressure; MAP: Mean arterial pressure; O_2_: Oxygen; HCO_3_: Bicarbonate; PaCO_2_: Partial arterial carbon dioxide pressure; RBS: Random blood sugar; Na: Sodium; K: Potassium; Mg: Magnesium; AST: Aspartate aminotransferase, ALT: Alanine aminotransferase.
